# Supplementary material for: Single Photon Randomness based on a Defect Center in Diamond
Source: Sci Rep. 2019 Dec 5;9:18474. doi: 10.1038/s41598-019-54594-0 (PMC6895230; doi:10.1038/s41598-019-54594-0)
Supplement: Supplementary file 1 — Supplermentary material [file 41598_2019_54594_MOESM1_ESM.pdf]

Supplementary material to:
Single Photon Randomness based on a Defect Center in Diamond

Xing Chen, Johannes Greiner, Jörg Wrachtrup and Ilja Gerhardt

Contents

1 Three Level System **2**

2 Data Post-processing **3**

2.1 Tuples . . . 3

2.2 The deduction of the conditional min-entropy . . . 4

2.3 Error bound of the entropy . . . 6

2.4 Fraction of single photon events . . . 7

2.5 The uncertainty of the classical limit line . . . 8

2.6 Two-universal hashing and the 11.5σ error bound . . . 10

3 Experimental results **11**

References **12**

This supplementary material provides details about some of the concepts and equations used in the main text. In the first section, the basic equations of the three level system and the photon statistics are introduced. In the second section, the data post-processing methods are described, including the derivation of the conditional min-entropy, the error bound of the conditional min-entropy, and two-universal hashing. In the third section, our experimental data is analysed.

1 Three Level System

The single photon source in our experimental setup is based on a single nitrogen-vacancy (NV) centre. This can be described as a three-level system. Please refer to Fig. S1 for a naming of the levels. The rate equations for this three level system are

$$\dot{\rho_1} = -k_{12}\rho_1 + k_{21}\rho_2 + k_{31}\rho_3 \ ,$$
$$\dot{\rho_2} = k_{12}\rho_1 - (k_{21} + k_{23})\rho_2 \ ,$$
$$\dot{\rho_3} = k_{23}\rho_2 - k_{31}\rho_3 \ .$$

where ρ_i denotes the population of each state, and $\dot{\rho_i}$ indicates the time derivative of ρ_i, k_{12} is the pumping rate, the other k_{ij} are the decay rates. Without excitation power, the NV centre is in its ground state, so the initial conditions are $\rho_1 = 1, \rho_2 = \rho_3 = 0$. By solving this differential equation system, we get $\rho_1(\tau), \rho_2(\tau), \rho_3(\tau)$. The auto-correlation function $g^{(2)}$ is defined as

$$g^{(2)}(\tau) = \frac{\rho_2(\tau)}{\rho_2(\infty)} \ . \qquad \text{(S1.1)}$$

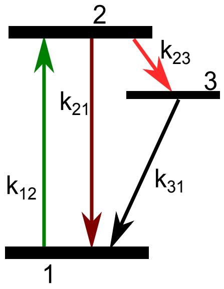

Figure S1: Three level system. The ground state is 1, the excited state is 2, and 3 is a meta-stable state.

The average fluorescence rate under continuous wave(CW) excitation is

$$F = \rho_2(\infty)k_{21} \ .$$

The different parameters used to fit the experimental saturation curve, as shown in Fig.2a in

the main text, are

$$
\begin{aligned}
k_{12} &= 0.77601 \text{ mW}^{-1} P_{\text{in}} \text{ ns}^{-1} \ , \\
k_{21} &= \frac{1}{16.08} \text{ ns}^{-1} \ , \\
k_{23} &= \left(\frac{1}{30.97} \text{ mW}^{-1} P_{\text{in}} + \frac{1}{1000} \right) \text{ ns}^{-1} \ , \\
k_{31} &= \left(\frac{1}{78.37} \text{mW}^{-1} P_{\text{in}} + \frac{1}{471.7} \right) \text{ ns}^{-1} \ .
\end{aligned}
$$

where P_{in} is the excitation power, unit is mW, and refers to the power at the entrance of the microscope objective. Theoretically, k_{12} equals P_{in}, but realistically, the excitation power cannot be used without loss, and that is why there is an efficiency parameter between k_{12} and P_{in}.

The relationship between the background noise events and the excitation power P_{in} is considered as linear, and their relationship is found to be

$$
BG_{\text{rate}} = 5.2 \times 10^6 \text{ mW}^{-1} P_{\text{in}} \text{ s}^{-1} \ .
$$

With background noise, the total photon events rate F_{bg} is

$$
\begin{aligned}
F_{\text{bg}} &= F + BG_{\text{rate}} \\
&= \rho_2(\infty) k_{21} + 5.2 \times 10^6 \text{ mW}^{-1} P_{\text{in}} \text{ s}^{-1} \ .
\end{aligned}
$$

The overall efficiency amounts to approximately 0.779% in our setup, this value denotes the ratio of detected photon events and the total photons events F_{bg}. This overall efficiency is affected by multiple parameters, including the transmission efficiencies of the lens, the reflection coefficients of the mirrors, and the detection efficiencies of the detectors.

Our data was recorded at $P_{\text{in}} = 26$mW, and according to the above equations, $F_{\text{bg}} = 1.28 \times 10^7 \text{s}^{-1}$. Since the overall efficiency here is 0.779%, the detection rate is about

$$
1.28 \times 10^7 \text{s}^{-1} \times 0.779\% = 99.7 \text{ kcps} \ .
$$

From S1.1 and [1], the equation for $g^{(2)}_{\text{fit}}(\tau)$ is derived as

$$
g^{(2)}_{\text{fit}}(\tau) = 1 - 1.06678 e^{-8.66533 \times 10^7 |\tau| \text{s}^{-1}} + 0.178378 e^{-2.00826 \times 10^6 |\tau| \text{s}^{-1}} \ . \tag{S1.2}
$$

$g^{(2)}_{\text{fit}}(\tau)$ is the formula of anti-bunching curve that fits our experimental data.

2 Data Post-processing

When we post-process the experimental data, there are four tuples 00, 01, 10, 11, and their corresponding probabilities are p(00), p(01), p(10), p(11).

2.1 Tuples

To calculate the conditional probabilities on a given set of experimental data, we count these tuple events in an overlapping fashion. This means, for example, for a number string like

010, when the tuples are overlapping, this string contains tuples 01 and 10; in the case of non-overlapping counting, this string only contains one tuple: 01.

The number of the overlapped tuples is calculated in the following way. Suppose we have a random string with N bits, when this random string is perfectly balanced, we should have $N(0) = N(1)$, this means that N at least should be an even number. Also, in a perfectly balanced random string, the number of tuples should satisfy two conditions: $N(00) = N(01) = N(10) = N(11)$ and $N(00)+N(01)+N(10)+N(11) = N$. For a random string with N bits, in order to satisfy $N(00) = N(01) = N(10) = N(11)$, N must be a number which can be divided by 4.

For any perfectly balanced random string, $N(00) + N(01) + N(10) + N(11) = N$ cannot be satisfied by a finite N, because for any finite N, the total number of tuples always adds up to $N-1$ instead of N. In order to make $N(00)+N(01)+N(10)+N(11) = N$, when we calculate the number of overlapped tuples, we put the last bit n_l and the first bit n_f together to get an additional tuple $n_l n_f$, and by this way the condition $N(00) + N(01) + N(10) + N(11) = N$ will be fulfilled. For example, for a random string like 1110101000001110, the length of this string is $N = 16$, while $N(00) = 4, N(01) = 3, N(10) = 4, N(11) = 4$, the total number of tuples is 15. When we put the last bit 0 and the first bit 1 together as a new tuple 01, it will make $N(01) = 4$, and they sum up to the desired number 16. We consider this as the optimal way to treat measured data and to calculate the frequencies of the single and double events.

2.2 The deduction of the conditional min-entropy

In this subsection, the conditional min-entropy mentioned in the main text is calculated in detail. The conditional min-entropy is defined as

$$H_\infty(X|Y) = -\log_2\big(\sum_y p(y)\max_x\{p(x|y)\}\big) \ .$$

where x and y are two subsequent random bits in a given random string.
In our case, $\{X, Y\} \in \{0, 1\}$, subsequently the conditional min-entropy is

$$\begin{aligned} H_\infty(X|Y) &= -\log_2\big(\sum_y p(y)\max_x\{p(x|y)\}\big) \\ &= -\log_2\big(p(0)\max\{p(0|0), p(1|0)\} + p(1)\max\{p(0|1), p(1|1)\}\big) \ . \end{aligned} \tag{S2.1}$$

For the convenience of description, the detector in the transmitted arm is named as detector A, and the detector in the reflected arm is named as detector B. Next, we deduce all the conditional probabilities from the experimental parameters. We note that this does not describe the true probabilities, but reflects the frequencies of the occurring singles and tuples.

Take $p(A|A)$ as an example, $p(A|A)$ means the probability of a subsequent photon to be detected in detector A when detector A has already detected a previous photon event. Let η_A be the detection efficiency of detector A, $\tau^\text{A}_\text{dead}$ be the dead-time of detector A, and T be the transmission coefficient of the beam-splitter. When detector A clicked, it is in its

dead-time. $\int_0^{\tau_{\text{dead}}^{\text{A}}} g_{\text{fit}}^{(2)}(\tau)d\tau$ means the probability that the next incident photon is in the dead-time of detector A, then the probability of this incident photon outside its dead-time is $1-\int_0^{\tau_{\text{dead}}^{\text{A}}} g_{\text{fit}}^{(2)}(\tau)d\tau$. When the incident photon is outside the dead-time of detector A, it has probability T to be transmitted to detector A, and detector A has probability η_{A} to detect this photon, so $p(A|A)$ could be written as

$$p(A|A) = \big(1-\int_0^{\tau_{\text{dead}}^{\text{A}}} g_{\text{fit}}^{(2)}(\tau)d\tau\big)\eta_{\text{A}}T \ . \tag{S2.2}$$

Similarly, the parametric equation for $p(B|A)$ is

$$p(B|A) = \eta_{\text{B}}R\big(1-\underbrace{\eta_{\text{B}}R\int_0^{\frac{\tau_{\text{dead}}^{\text{B}}}{2}} g_{\text{fit}}^{(2)}(\tau)d\tau \int_0^{\frac{\tau_{\text{dead}}^{\text{B}}}{2}} g_{\text{fit}}^{(2)}(\tau)d\tau\big)}_{\text{probability that detector B is in its dead-time}} \ . \tag{S2.3}$$

where η_{B} is the detection efficiency of detector B, $\tau_{\text{dead}}^{\text{B}}$ is the dead-time of detector B, and R is the reflection coefficient. This gives us the equation of $p(B|A)$, which means when detector A detects a photon event, the probability of detector B detecting a subsequent photon event.

The formula in the underbrace means the probability of detector B is not in its dead-time when a photon shoots into the beam-splitter, this probability is an estimation, which is based on the assumption $\tau_{\text{dead}}^{\text{A}} \approx \tau_{\text{dead}}^{\text{B}}$. Before detector A clicks, the previous photon event may be on detector A or B. If it is on detector A, it will not affect the conditional probability $p(B|A)$, since when detector A clicks two times, detector B is ready to detect a photon event; if the previous photon event is on detector B, then after detector A's click, detector B still has a probability to be in its dead-time when the next photon comes into the beam-splitter. Inside the brace, the half of the dead-time of detector B is a simplified version of the above probability analysis, where $\eta_{\text{B}}R\int_0^{\frac{\tau_{\text{dead}}^{\text{B}}}{2}} g_{\text{fit}}^{(2)}(\tau)d\tau$ gives us the probability that the previous photon event fires on detector B within the half of the dead-time of detector B, and $\int_0^{\frac{\tau_{\text{dead}}^{\text{B}}}{2}} g_{\text{fit}}^{(2)}(\tau)d\tau$ is the probability that the next incident photon is inside the half of the dead-time of detector B.

$p(B|B)$ and $p(A|B)$ can be derived analogously.

The equation of $p(A)$ is $p(A) = r_{\text{A}}/r_{\text{total}}$, r_{A} is the click rate of detector A. r_{A} is defined as following

$$\begin{aligned} r_{\text{A}} =& \eta_{\text{A}}TI_{\text{in}} - \underbrace{\frac{(\eta_{\text{A}}TI_{\text{in}})}{2} \times \frac{(\eta_{\text{A}}TI_{\text{in}})}{2}\int_0^{\tau_{\text{dead}}^{\text{A}}} g_{\text{fit}}^{(2)}(\tau)d\tau}_{\text{rate of two clicks within the dead-time of detector A}} \\ =& \ \eta_{\text{A}}TI_{\text{in}} - \frac{(\eta_{\text{A}}TI_{\text{in}})^2\int_0^{\tau_{\text{dead}}^{\text{A}}} g_{\text{fit}}^{(2)}(\tau)d\tau}{4} \ . \end{aligned} \tag{S2.4}$$

where I_{in} is the rate of incident photon, and $\eta_{\text{A}}TI_{\text{in}}$ means the click rate when detector A would have no dead-time. The latter part of the equation is the probability of two subsequent events in detector A to have a time distance which is smaller than the dead-time of detector

A. For detector B, a similar equation of r_{B} is derived, we get

$$\begin{aligned} p(A) &= \frac{r_{\mathrm{A}}}{r_{\mathrm{A}}+r_{\mathrm{B}}} \\ &= \frac{\eta_{\mathrm{A}} T I_{\mathrm{in}} - \frac{(\eta_{\mathrm{A}} T I_{\mathrm{in}})^2 \int_0^{\tau_{\mathrm{dead}}^{\mathrm{A}}} g_{\mathrm{fit}}^{(2)}(\tau)d\tau}{4}}{\eta_{\mathrm{A}} T I_{\mathrm{in}} - \frac{(\eta_{\mathrm{A}} T I_{\mathrm{in}})^2 \int_0^{\tau_{\mathrm{dead}}^{\mathrm{A}}} g_{\mathrm{fit}}^{(2)}(\tau)d\tau}{4} + \eta_{\mathrm{B}} R I_{\mathrm{in}} - \frac{(\eta_{\mathrm{B}} R I_{\mathrm{in}})^2 \int_0^{\tau_{\mathrm{dead}}^{\mathrm{B}}} g_{\mathrm{fit}}^{(2)}(\tau)d\tau}{4}} \\ &= \frac{\eta_{\mathrm{A}} T - \frac{(\eta_{\mathrm{A}} T)^2 I_{\mathrm{in}} \int_0^{\tau_{\mathrm{dead}}^{\mathrm{A}}} g_{\mathrm{fit}}^{(2)}(\tau)d\tau}{4}}{\eta_{\mathrm{A}} T - \frac{(\eta_{\mathrm{A}} T)^2 I_{\mathrm{in}} \int_0^{\tau_{\mathrm{dead}}^{\mathrm{A}}} g_{\mathrm{fit}}^{(2)}(\tau)d\tau}{4} + \eta_{\mathrm{B}} R - \frac{(\eta_{\mathrm{B}} R)^2 I_{\mathrm{in}} \int_0^{\tau_{\mathrm{dead}}^{\mathrm{B}}} g_{\mathrm{fit}}^{(2)}(\tau)d\tau}{4}} . \end{aligned} \tag{S2.5}$$

With all the above equations, the parametric expression of $H_\infty(X|Y)$ could be deduced.

2.3 Error bound of the entropy

In this subsection, the error bound of the conditional min-entropy is given. Let us mention some properties of the error bound. Since $p(A) + p(B) = 1$ is always fulfilled, we have $\Delta_{p(A)} = -\Delta_{p(B)}$. Also, for conditional probabilities $p(A|A), p(B|A), p(A|B), p(B|B)$, $p(A|A) + p(B|A) = 1$ and $p(A|B) + p(B|B) = 1$, this means $\Delta_{p(A|A)} = -\Delta_{p(B|A)}$ and $\Delta_{p(A|B)} = -\Delta_{p(B|B)}$. Since $p(AB) = p(BA)(p(BA) = p(AB)$ is satisfied under the condition that the experimental devices does not change over time), and $\Delta_{p(A)} = -\Delta_{p(B)}$, it is easy to derive the relationship $\Delta_{p(A|A)} = -\Delta_{p(B|A)} = \Delta_{p(A|B)} = -\Delta_{p(B|B)}$.

The conditional min-entropy in our case is defined in S2.1. Since $p(BA) = p(AB)$,

$$H_\infty(X|Y) = -\log_2\big(\max\{p(A) - p(AB), p(AB)\} + \max\{p(AB), 1 - p(A) - p(AB)\}\big) . \tag{S2.6}$$

There are four different conditions for $H_\infty(X|Y)$

$$H_\infty(X|Y) = \begin{cases} -\log_2(p(A)) & p(A) - p(AB) \geq p(AB) \quad and \quad p(AB) \geq 1 - p(A) - p(AB) , \\ -\log_2(p(B)) & p(A) - p(AB) \leq p(AB) \quad and \quad p(AB) \leq 1 - p(A) - p(AB) , \\ -\log_2(2p(AB)) & p(A) - p(AB) \leq p(AB) \quad and \quad p(AB) \geq 1 - p(A) - p(AB) , \\ -\log_2(1 - 2p(AB)) & p(A) - p(AB) \geq p(AB) \quad and \quad p(AB) \leq 1 - p(A) - p(AB) . \end{cases} \tag{S2.7}$$

No matter which condition $H_\infty(X|Y)$ is in, there is only one variable in it. Then a more conservative conditional min-entropy could be written as

$$H_\infty(X|Y) = -\log_2(f(p) + \Delta_{f(p)}) \tag{S2.8}$$

where $f(p) = \max\{p(A), p(B), 2p(AB), 1 - 2p(AB)\}$.

The equation of $p(A)$ is Eqn. S2.5, $p(A)$ is affected by the transmission coefficient T, the rate of incident photon I_{in}, the detection efficiency η_{A}, η_{B}, and the dead-time τ_{dead}^{A}, τ_{dead}^{B} of the two detectors. The error bound of each parameter is, δ_T, $\delta_{\eta_{\mathrm{A}}}$,$\delta_{\eta_{\mathrm{B}}}$, $\delta_{\tau_{\mathrm{dead}}^{A}}$,$\delta_{\tau_{\mathrm{dead}}^{B}}$, and $\delta_{I_{\mathrm{in}}}$.

According to the error propagation, the error bound of $p(A)$ is

$$\Delta_{p(A)} = \Big((\frac{\partial p(A)}{\partial T}\delta_T)^2 + (\frac{\partial p(A)}{\partial \eta_{\mathrm{A}}}\delta_{\eta_{\mathrm{A}}})^2 + (\frac{\partial p(A)}{\partial \eta_{\mathrm{B}}}\delta_{\eta_{\mathrm{B}}})^2 + (\frac{\partial p(A)}{\partial \tau^A_{\mathrm{dead}}}\delta_{\tau^A_{\mathrm{dead}}})^2 + (\frac{\partial p(A)}{\partial \tau^B_{\mathrm{dead}}}\delta_{\tau^B_{\mathrm{dead}}})^2 + (\frac{\partial p(A)}{\partial I_{\mathrm{in}}}\delta_{I_{\mathrm{in}}})^2 \Big)^{\frac{1}{2}} . \tag{S2.9}$$

The equation of $p(AB)$ is $p(AB) = p(A)p(B|A)$. From S2.3 and S2.5, we know that multiple parameters affect $p(AB)$, including τ^A_{dead}, τ^B_{dead}, η_{A}, η_{B}, T, and I_{in}, similarly, $\Delta_{p(AB)}$ is

$$\Delta_{p(AB)} = \Big((\frac{\partial p(AB)}{\partial T}\delta_T)^2 + (\frac{\partial p(AB)}{\partial \eta_{\mathrm{A}}}\delta_{\eta_{\mathrm{A}}})^2 + (\frac{\partial p(AB)}{\partial \eta_{\mathrm{B}}}\delta_{\eta_{\mathrm{B}}})^2 + (\frac{\partial p(AB)}{\partial \tau^A_{\mathrm{dead}}}\delta_{\tau^A_{\mathrm{dead}}})^2 + (\frac{\partial p(AB)}{\partial \tau^B_{\mathrm{dead}}}\delta_{\tau^B_{\mathrm{dead}}})^2 + (\frac{\partial p(AB)}{\partial I_{\mathrm{in}}}\delta_{I_{\mathrm{in}}})^2 \Big)^{\frac{1}{2}} . \tag{S2.10}$$

Then from Eqn. S2.8, S2.9, S2.10, the conservative $H_\infty(X|Y)$ could be calculated.

For the second model, there is one more parameter p_e, which represents the probability of detecting an uncorrelated background noise event. The conditional min-entropy for the second model is

$$\begin{aligned} H_\infty(X|Y) &= -\log_2\Big(p_e + (1-p_e)(\sum_y p(y)\max_x\{p(x|y)\})\Big) \\ &= -\log_2\Big(p_e + (1-p_e)f(p)\Big) . \end{aligned} \tag{S2.11}$$

where $f(p) = \max\{p(A), p(B), 2p(AB), 1-2p(AB)\}$. A more conservative $H_\infty(X|Y)$ for this model is

$$H_\infty(X|Y) = -\log_2\big(p_{eq} + \Delta_{p_{eq}}\big) \tag{S2.12}$$

where $p_{eq} = p_e + (1-p_e)f(p)$, then $\Delta_{p_{eq}} = \sqrt{(\frac{\partial p_{eq}}{\partial p_e}\Delta_{p_e})^2 + (\frac{\partial p_{eq}}{\partial f(p)}\Delta_{f(p)})^2}$, where $\Delta_{f(p)}$ is derived from S2.9 and S2.10, and Δ_{p_e} is shown in S2.14.

2.4 Fraction of single photon events

In the second model, regarding the fraction of the single photon events, we assume that the background noise is uncorrelated, and the single photon events are randomly mixed with the uncorrelated background noise. In this scenario, let the fraction of single photon events be s, then the fraction of the uncorrelated background noise is $1-s$. The fraction of single photon events is [1]

$$s = \sqrt{1 - g^{(2)}_{\mathrm{fit}}(0)} . \tag{S2.13}$$

Of course, the deviation of the $g^{(2)}_{\mathrm{fit}}(0)$ from 0 does not mean background noise all the time, it may also be caused by multiple emitters [2]. The multiple equally bright emitters will change $g^{(2)}_{\mathrm{fit}}(0)$ to $1 - \frac{1}{n}$, where n is the number of emitters. For example, when $g^{(2)}_{\mathrm{fit}}(0) = 0.5$, it could be caused by background noise or two emitters. When it is caused by two emitters, all the events are from single photon sources; when it is caused by background noise, from S2.13, we

know that only 70.7% events are single photon events. From a conservative consideration, as long as $g_{\text{fit}}^{(2)}(0)$ deviates from 0, we treat it as the result of background noise, instead of some extra emitters.

Note that the uncertainty of $g_{\text{fit}}^{(2)}(0)$ mentioned above will again affect the fraction of background noise, thus affect p_e. Since $p_e = 1-s = 1-\sqrt{1-g_{\text{fit}}^{(2)}(0)}$, according to the propagation of uncertainty, the uncertainty of p_e is

$$\Delta_{p_e} = \frac{1}{2\sqrt{1-g_{\text{fit}}^{(2)}(0)}}\Delta_{g_{\text{fit}}^{(2)}(0)} \ . \tag{S2.14}$$

where $\Delta_{g_{\text{fit}}^{(2)}(0)}$ is derived in the following(see Eqn. S2.24).

2.5 The uncertainty of the classical limit line

In the third model, the extractable quantum randomness in the raw data is determined by the single photon start-stop event count rate under the classical limit line [2–4]

$$\begin{aligned}
r_{\text{rand}} &= r_{\text{A}}\sqrt{1-g_{\text{fit}}^{(2)}(0)} \times r_{\text{B}}\sqrt{1-g_{\text{fit}}^{(2)}(0)} \times \int_{-t}^{t} g_{\text{fit}}^{(2)}(\tau)d\tau \\
&= (1-g_{\text{fit}}^{(2)}(0)) \times (\eta_{\text{A}}TI_{\text{in}} - \frac{(\eta_{\text{A}}TI_{\text{in}})^2\int_0^{\tau_{\text{dead}}^{\text{A}}} g_{\text{fit}}^{(2)}(\tau)d\tau}{4})(\eta_{\text{B}}RI_{\text{in}} - \frac{(\eta_{\text{B}}RI_{\text{in}})^2\int_0^{\tau_{\text{dead}}^{\text{B}}} g_{\text{fit}}^{(2)}(\tau)d\tau}{4}) \\
&\quad \times \int_{-t}^{t} g_{\text{fit}}^{(2)}(\tau)d\tau \\
&= (1-g_{\text{fit}}^{(2)}(0)) \times (\eta_{\text{A}}T - \frac{(\eta_{\text{A}}T)^2I_{\text{in}}\int_0^{\tau_{\text{dead}}^{\text{A}}} g_{\text{fit}}^{(2)}(\tau)d\tau}{4})(\eta_{\text{B}}R - \frac{(\eta_{\text{B}}R)^2I_{\text{in}}\int_0^{\tau_{\text{dead}}^{\text{B}}} g_{\text{fit}}^{(2)}(\tau)d\tau}{4}) \\
&\quad \times I_{\text{in}}^2\int_{-t}^{t} g_{\text{fit}}^{(2)}(\tau)d\tau \ .
\end{aligned} \tag{S2.15}$$

where t satisfies $g_{\text{fit}}^{(2)}(t) = 1$. And the quantum fraction of the raw bits is defined as $r_{\text{rand}}/r_{\text{total}}$, then p_c, the classical noise probability, is $p_c = 1 - r_{\text{rand}}/r_{\text{total}}$. Under the classical limit line, the events 0-1 is taken as random bit **0**, and 1-0 is taken as **1**, the conditional min-entropy in this case is defined as

$$\begin{aligned}
H_\infty(\mathbf{X}|\mathbf{Y}) &= -\log_2\Big(p_c + (1-p_c)\big(\max\{p(\mathbf{00}), p(\mathbf{01})\} + \max\{p(\mathbf{10}), p(\mathbf{11})\}\big)\Big) \\
&= -\log_2\Big(p_c + (1-p_c)f(\mathbf{p})\Big) \ .
\end{aligned} \tag{S2.16}$$

where $f(\mathbf{p}) = \max\{p(\mathbf{0}), p(\mathbf{1}), 2p(\mathbf{01}), 1-2p(\mathbf{01})\}$.

For the convenience of description, without losing generality, we associate event pair "AB" to random bit **0**, and "BA" to **1**. For probabilities $p(\mathbf{0})$ and $p(\mathbf{1})$, there are two different situations. The first situation: when t is larger than the half of the dead-time of the detectors,

we have

$$p(\mathbf{0}) = p(AB) = p(A)\eta_{\rm B}R\Big(\int_0^t g^{(2)}_{\rm fit}(\tau)d\tau - \eta_{\rm B}R\int_0^{\frac{\tau^{\rm B}_{\rm dead}}{2}} g^{(2)}_{\rm fit}(\tau)d\tau \times \underbrace{\int_0^{\frac{\tau^{\rm B}_{\rm dead}}{2}} g^{(2)}_{\rm fit}(\tau)d\tau}_{\text{incident photon within } \tau^{\rm B}_{\rm dead}/2.}\Big) \ . \tag{S2.17}$$

The probability here is very similar to S2.3, except we only consider short-time related photon events in this situation, so we replace '1' in S2.3 with $\int_0^t g^{(2)}_{\rm fit}(\tau)d\tau$ here, where $p(A)$ is in S2.5. The other situation is when t is smaller than the half of the dead-time of each detector, we need to change the formula inside the underbrace to $\int_0^t g^{(2)}_{\rm fit}(\tau)d\tau$, then

$$p(\mathbf{0}) = p(AB) = p(A)\eta_{\rm B}R\int_0^t g^{(2)}_{\rm fit}(\tau)d\tau\big(1 - \eta_{\rm B}R\int_0^{\frac{\tau^{\rm B}_{\rm dead}}{2}} g^{(2)}_{\rm fit}(\tau)d\tau\big) \ . \tag{S2.18}$$

The equation of $p(\mathbf{1})$ can be deduced in a similar way.

For the photon events under the classical limit line, the events pair $\mathbf{0}$ or $\mathbf{1}$ is are much less correlated than previous models, they can be treated as independent events, so we have

$$\begin{aligned} p(\mathbf{00}) &= p(\mathbf{0})p(\mathbf{0}) \ , \\ p(\mathbf{01}) &= p(\mathbf{0})p(\mathbf{1}) \ , \\ p(\mathbf{10}) &= p(\mathbf{1})p(\mathbf{0}) \ , \\ p(\mathbf{11}) &= p(\mathbf{1})p(\mathbf{1}) \ . \end{aligned} \tag{S2.19}$$

Next we calculate the conservative conditional min-entropy in this model, similar to the second model, we have

$$H_\infty(\mathbf{X}|\mathbf{Y}) = -\log_2\big(p_{cq} + \mathit{\Delta}_{p_{cq}}\big) \ . \tag{S2.20}$$

where $p_{cq} = p_c + (1-p_c)f(\mathbf{p})$, then $\mathit{\Delta}_{p_{cq}} = \sqrt{(\frac{\partial p_{cq}}{\partial p_c}\mathit{\Delta}_{p_c})^2 + (\frac{\partial p_{cq}}{\partial f(\mathbf{p})}\mathit{\Delta}_{f(\mathbf{p})})^2}$. From $p_c = 1 - r_{\rm rand}/r_{\rm total}$, we get

$$p_c = 1 - \frac{(1-g^{(2)}_{\rm fit}(0)) \times r_{\rm A} \times r_{\rm B} \times \int_{-t}^t g^{(2)}_{\rm fit}(\tau)d\tau}{r_{\rm A} + r_{\rm B}} \ . \tag{S2.21}$$

and the equation for $f(\mathbf{p})$ is in S2.18, S2.17 and S2.19. From the equations of p_c and $f(\mathbf{p})$, we can see that they are dependent on some same parameters, including the dead-time of the two detectors, the detection efficiencies, and the beam-splitter ratio etc. This means that they are not independent from each other, so $\mathit{\Delta}_{p_{cq}} \neq \sqrt{(\frac{\partial p_{cq}}{\partial p_c}\mathit{\Delta}_{p_c})^2 + (\frac{\partial p_{cq}}{\partial f(\mathbf{p})}\mathit{\Delta}_{f(\mathbf{p})})^2}$, $\mathit{\Delta}_{p_{cq}}$ should be derived directly from the experimental parameters

$$\begin{aligned} \mathit{\Delta}_{p_{cq}} = \Big(&(\frac{\partial p_c}{\partial T}\delta_T)^2 + (\frac{\partial p_c}{\partial \eta_{\rm A}}\delta_{\eta_{\rm A}})^2 + (\frac{\partial p_c}{\partial \eta_{\rm B}}\delta_{\eta_{\rm B}})^2 + (\frac{\partial p_c}{\partial \tau^A_{\rm dead}}\delta_{\tau^A_{\rm dead}})^2 + \\ &(\frac{\partial p_c}{\partial \tau^B_{\rm dead}}\delta_{\tau^B_{\rm dead}})^2 + (\frac{\partial p_c}{\partial I_{\rm in}}\delta_{I_{\rm in}})^2 + \frac{\partial p_c}{\partial t}\delta_t)^2\Big)^{\frac{1}{2}} \ . \end{aligned} \tag{S2.22}$$

where t satisfies $g^{(2)}_{\rm fit}(t) = 1$. Next we derive δ_t. In our case, δ_t is characterized by the classical limit line. The classical limit line is determined by the normalization factor of the experimental anti-bunching curve. The normalization factor $N_{\rm norm}$ is calculated by

$$N_{\rm norm} = r_{\rm A} r_{\rm B} \tau_{\rm rs} T_{\rm total}$$

where $\tau_{\rm rs}$ is the timing resolution of the start-stop event, $T_{\rm total}$ is the total integration time (the running time of the experiment). $N_{\rm norm}$ can be determined by multiple parameters, including the detection efficiency and dead-time of each detector, and the reflection and transmission coefficients. According to the propagation of uncertainty, we get the uncertainty of $N_{\rm norm}$

$$\begin{aligned}\varDelta_{\rm norm} = \Bigg(&(\frac{\partial N_{\rm norm}}{\partial R}\delta_R)^2 + (\frac{\partial N_{\rm norm}}{\partial \eta_{\rm A}}\delta_{\eta_{\rm A}})^2 + \\ &(\frac{\partial N_{\rm norm}}{\partial \eta_{\rm B}}\delta_{\eta_{\rm B}})^2 + (\frac{\partial N_{\rm norm}}{\partial I_{\rm in}}\delta_{I_{\rm in}})^2 + \\ &(\frac{\partial N_{\rm norm}}{\partial \tau^{\rm A}_{\rm dead}}\delta_{\tau^{\rm A}_{\rm dead}})^2 + (\frac{\partial N_{\rm norm}}{\partial \tau^{\rm B}_{\rm dead}}\delta_{\tau^{\rm B}_{\rm dead}})^2\Bigg)^{\frac{1}{2}} .\end{aligned}$$

Then the uncertainty of the classical limit line (i.e. $g^{(2)}_{\rm fit}(\tau) = 1$) amounts to

$$\varDelta_1 = 1 \times \frac{\varDelta_{\rm norm}}{N_{\rm norm}} . \tag{S2.23}$$

and the uncertainty of the background line $g^{(2)}_{\rm fit}(0)$ is

$$\varDelta_{g^{(2)}_{\rm fit}(0)} = g^{(2)}_{\rm fit}(0)\frac{\varDelta_{\rm norm}}{N_{\rm norm}} . \tag{S2.24}$$

From the uncertainty of classical limit line, $\delta_t = t - t'$ can be deduced, where t' satisfies the equation $g^{(2)}_{\rm fit}(t') = 1 - \varDelta_1$. Then $\varDelta_{p_{cq}}$ can be derived.

2.6 Two-universal hashing and the 11.5σ error bound

Two-universal hashing can be used as a randomness extractor [5]. The main idea of this extractor is explained in [6]: For a n bits random string X, we want to extract a random string Y, which is k bits long, $k < n$. The quality of a random extractor is quantified by the probability that the output random string Y deviates from a perfect uniformly distributed k-bit string.

Two-universal hashing can be simply done by a bit-matrix-vector multiplication with a seed m (a random matrix with dimension $n \times k$)

$$y_i = \sum_{j=1}^{n} m_{i,j} x_j .$$

where $y_i \in Y$, and $x_j \in X$.
Let ϵ be the deviation, and H be the entropy in the input string X, then with two-universal

hashing, the deviation ϵ is bound by [5, 7]

$$\epsilon = 2^{-(Hn-k)/2} \ .$$

In our case, the error bound ϵ is decided to be 2^{-100} or 10^{-30} [6]. The physical meaning of this bound is that in the age of the universe, even when one million different random strings are hashed by this two-universal hashing, we cannot observe any deviation from a perfect uniform randomness in the output string.

The 11.5σ error bound mentioned below can be derived by the following Gaussian distribution

$$1 - \int_{-m\sigma}^{m\sigma} \frac{1}{\sqrt{2\pi\sigma^2}} e^{-\frac{x^2}{2\sigma^2}} dx = 2^{-100} \ .$$

the solution of m is $m = \sqrt{2}\mathrm{erf}^{-1}\left(\frac{2^{100}-1}{2^{100}}\right) \approx 11.5$, where erf is the Error Function.

3 Experimental results

In this section, some calculation details of the experiment results are described. First we estimate the 1σ error bound of the experimental devices. Reasonably, we have the following assumption: the error bound of the beam-splitter is $\delta_R = 0.004$, the error bound of the two detectors' detection efficiencies is $\delta_{\eta_A} = \delta_{\eta_B} = 0.01$, the error bound of dead-time is $\delta_{\tau^A_{\mathrm{dead}}} = \delta_{\tau^B_{\mathrm{dead}}} = 10$ ns, and the error bound of the incident photon rate is $\delta_{I_{\mathrm{in}}} = \sqrt{I_{\mathrm{in}}}$.

The values of the experimental parameters are estimated as

$\tau^{\mathrm{A}}_{\mathrm{dead}}$	η_{A}	T	$\tau^{\mathrm{B}}_{\mathrm{dead}}$	η_{B}	R	I_{in}
43.5ns	60%	0.39	42.9ns	60%	0.61	166 kcps

Table 1: **The parameter value of experimental devices**

For the first model, according to S2.5 and S2.10, $p(1) = 0.6100$, and $p(10) = 0.2379$, considering the different conditions in S2.7, the output conditional min-entropy per raw bit is 0.7132 bits, when introducing the 11.5σ error bound, from S2.8, the conservative conditional min-entropy is

$$H_\infty(X|Y) = -\log_2\big(p(1) + 11.5\mathit{\Delta}_{p(1)}\big) = 0.5559 \ .$$

This means that the conservative randomness per raw bit is 0.5559 bits, where $\mathit{\Delta}_{p(1)} = 0.006$ is from S2.9. For this model, the experimental data gives us a value of $H_\infty(X|Y) = 0.7128$, which is within the given error bound.

With the conservative 11.5σ error bound, by two-universal hashing, approximate 3.10×10^{10} unbiased random bits can be extracted from the raw random bits, and the unbiased random number generation speed is 3.10×10^{10}bits/608125s $= 5.1 \times 10^4$ bits per second.

In the second model, by limiting the raw random data to single photon events, we exclude the uncorrelated background noise from the total photon events. According to this model,

we get $p(1) = 0.6100$, $g^{(2)}_{\rm fit}(0) = 0.1116$, and the fraction of the uncorrelated background noise $p_e = 1 - \sqrt{1 - 0.1116} = 0.0575$. From S2.11, the output quantum randomness per raw bit is 0.6612 bits. From S2.12, considering the 11.5σ error bound, the conservative conditional min-entropy is

$$H_\infty(X|Y) = -\log_2\left(p_{eq} + 11.5\varDelta_{p_{eq}}\right) = 0.5168 \ .$$

where $p_{eq} = 0.6324, \varDelta_{p_{eq}} = 0.0058$ can be calculated from 2.3. In the experimental data, $g^{(2)}_{\rm fit}(0) = 0.1114$, and $p(1) = 0.6101$, using S2.11, the extractable quantum randomness per raw bit is 0.6610 bits, which is covered by the 11.5σ error bound. Then with the strict 11.5σ error bound, the total quantum random number in the raw data is 2.88×10^{10} bits, and the generation speed is about 2.88×10^{10}bits/608125s $= 4.74 \times 10^4$ bits per second.

In the third model, a more strict quantum randomness extraction method is introduced. In most cases, when $g^{(2)}_{\rm fit}(0) < 1$ the photons are non-classical [2–4], and it is assumed that only single photons could reach the domain of $g^{(2)}_{\rm fit}(0) < 1$. To limit our photon events to single photons, we only consider events that are below the classical limit line and above the background noise line.

From S2.21, $p_c = 0.99939$. Solving $g^{(2)}_{\rm fit}(t) = 1$, we have $t = 21.13$ ns, since it is smaller than the half of the dead-time of two detectors, according to S2.18, we have $p(\mathbf{0}) = 0.500051$ and $p(\mathbf{1}) = 0.499949$. From S2.19 and S2.16, the output quantum randomness per raw bit is $H_\infty(\mathbf{X}|\mathbf{Y}) = 4.396 \times 10^{-4}$ bits. Considering the 11.5σ error bound, from S2.20 and S2.22, we compute the extractable quantum randomness per raw bit is

$$H_\infty(\mathbf{X}|\mathbf{Y}) = -\log_2\left(p_{cq} + 11.5\varDelta_{p_{cq}}\right) = 3.746 \times 10^{-4} \ .$$

In the experimental data, the data below the classical limit line is short-time related. By post-processing, the total events below the classical limit line is 32607956 bits, these bits are generated in a time without refocusing, which is 558000 seconds, then the tuple events per second is about 58.43 bits. The generation speed for the raw bits is about 100 kcps, thus $58.43/100000 \approx 5.843 \times 10^{-4}$ is the fraction of quantum random bits in the raw bits, the rest is considered as classical noise, $p_c = 1 - 5.843 \times 10^{-4} = 0.999416$. In these quantum random bits, we have 16305447 bits which are $\mathbf{0}$, and 16302509 bits $\mathbf{1}$, $p(\mathbf{0}) = 0.500045, p(\mathbf{1}) = 0.499955$. Then the extractable quantum randomness from per raw bit is 4.211×10^{-4}, which does not exceed the 11.5σ error bound.

With the given error bound, the number of quantum random bits in the raw data is 2.09×10^7 bits, the generation speed of these quantum random bits is 2.09×10^7bits/608125s =34.37 bits per second.

References

[1] Rosa Brouri, Alexios Beveratos, Jean-Philippe Poizat, and Philippe Grangier. Photon antibunching in the fluorescence of individual color centers in diamond. *Opt. Lett.*, 25(17): 1294–1296, Sep 2000. doi: 10.1364/OL.25.001294. URL `http://ol.osa.org/abstract.cfm?URI=ol-25-17-1294`.

[2] Rodney Loudon. *The quantum theory of light*. OUP Oxford, 2000.

[3] H. Paul. Photon antibunching. *Rev. Mod. Phys.*, 54:1061–1102, Oct 1982. doi: 10.1103/RevModPhys.54.1061. URL https://link.aps.org/doi/10.1103/RevModPhys.54.1061.

[4] Mark Fox. *Quantum Optics: An Introduction*. Oxford University Press, 2006.

[5] Daniela Frauchiger, Renato Renner, and Matthias Troyer. True randomness from realistic quantum devices. *ArXiv*, 2013. URL http://arxiv.org/abs/1311.4547.

[6] M. Troyer and R. Renner. A randomness extractor for the quantis device. *Internal Report*, pages 1–7, 2012. URL http://marketing.idquantique.com/acton/attachment/11868/f-004d/1/-/-/-/-/quantis-rndextract-techpaper.pdf.

[7] Marco Tomamichel, Christian Schaffner, Adam Smith, and Renato Renner. Leftover hashing against quantum side information. *IEEE Transactions on Information Theory*, 57(8):5524–5535, 2011. URL https://ieeexplore.ieee.org/document/5961850.
